# Studying Rare Movement Disorders: From Whole-Exome Sequencing to New Diagnostic and Therapeutic Approaches in a Modern Genetic Clinic

**DOI:** 10.3390/biomedicines12122673

**Published:** 2024-11-23

**Authors:** Luca Marsili, Kevin R. Duque, Jesus Abanto, Nathaly O. Chinchihualpa Paredes, Andrew P. Duker, Kathleen Collins, Marcelo Miranda, M. Leonor Bustamante, Michael Pauciulo, Michael Dixon, Hassan Chaib, Josefina Perez-Maturo, Emily J. Hill, Alberto J. Espay, Marcelo A. Kauffman

**Affiliations:** 1 Gardner Family Center for Parkinson’s Disease and Movement Disorders, Department of Neurology, University of Cincinnati, Cincinnati, OH 45219, USA; duqueykr@ucmail.uc.edu (K.R.D.); abantojs@ucmail.uc.edu (J.A.); chinchny@ucmail.uc.edu (N.O.C.P.); dukeraa@ucmail.uc.edu (A.P.D.); hill2e9@ucmail.uc.edu (E.J.H.); espayaj@ucmail.uc.edu (A.J.E.); 2 Division of Human Genetics, Department of Pediatrics, Children’s Hospital Medical Center, Cincinnati, OH 45229, USA; kathleen.collins2@cchmc.org (K.C.); mike.pauciulo@cchmc.org (M.P.); michael.dixon@cchmc.org (M.D.); hassan.chaib@cchmc.org (H.C.); 3 Clinica MEDS, Santiago 7690727, Chile; marcelomirandac@gmail.com (M.M.); mbustamante@uchile.cl (M.L.B.); 4 Fundación Diagnosis, Santiago 7500967, Chile; 5 Human Genetics Program, Biomedical Sciences Institute, Faculty of Medicine, Universidad de Chile, Santiago 8380000, Chile; 6 Department of Pediatrics, University of Cincinnati College of Medicine, Cincinnati, OH 45221, USA; 7 Neurogenetics Unit, Hospital JM Ramos Mejía, Buenos Aires C1221ADC, Argentina; josefinaperezmaturo@gmail.com (J.P.-M.); marcelokauffman@gmail.com (M.A.K.); 8 Precision Medicine and Clinical Genomics Group, Translational Medicine Research Institute—CONICET, Faculty of Biomedical Sciences, Universidad Austral, Buenos Aires C1221ADC, Argentina

**Keywords:** genetics, parkinsonism, ataxia, whole-exome sequencing, long-read sequencing

## Abstract

**Background:** Rare movement disorders often have a genetic etiology. New technological advances have increased the odds of achieving genetic diagnoses: next-generation sequencing (NGS) (whole-exome sequencing—WES; whole-genome sequencing—WGS) and long-read sequencing (LRS). In 2017, we launched a WES program for patients with rare movement disorders of suspected genetic etiology. We aim to describe the accumulated experience of a modern movement disorder genetic clinic, highlighting how different available genetic tests might be prioritized according to the clinical phenotype and pattern of inheritance. **Methods:** Participants were studied through WES analysis. Descriptive statistics, including the mean, standard deviation, counts, and percentages, were used to summarize demographic and clinical characteristics in all subjects and with each type of result [pathogenic or likely pathogenic, variants of uncertain significance (VUS), negative]. **Results:** We studied 88 patients (93.2% Caucasian, 5.72% African American, and 1.08% Hispanic or Latino). After excluding six family members from four index participants, the diagnostic yield of WES reached 27% (22/82 probands). The age at onset was significantly lower in patients with pathogenic/likely pathogenic variants. The most common clinical phenotypes were ataxia and parkinsonism. Dystonia, ataxia, leukoencephalopathy, and parkinsonism were associated with most genetic diagnoses. **Conclusions:** We propose a comprehensive protocol with decision tree testing for WGS and LRS, a return of results, and a re-analysis of inconclusive genetic data to increase the diagnostic yield of patients with rare neurogenetic disorders.

## 1. Introduction

Rare movement disorders are, by definition, a number of conditions genetically driven that may manifest with either isolated or combined phenotypes including, but not limited to, ataxia, parkinsonism, dystonia, chorea, tremors, and other rarer phenotypes [1]. Genetic variants associated with rare movement disorders frequently remain undiagnosed for long periods of time. This results in patients’ frustration, high costs due to inconclusive medical evaluations, and missed opportunities to consider available treatments or participate in trials of promising experimental interventions [2,3]. Thus, early genetic diagnosis is increasingly important. Unfortunately, genetic testing is typically not covered by insurance and is often not affordable through traditional commercial avenues [2,3]. In addition, rapid advances in our understanding of the biological mechanisms responsible for rare genetic disorders have enabled the development of specific treatments for selected conditions in line with the principles of precision medicine [4,5,6,7]. In this view, the improvement of our knowledge of genetic variants in variable neurologic conditions is critical in the setting of a modern movement disorder genetic clinic [8].

Next-generation sequencing (NGS) and, more specifically, whole-exome sequencing (WES) have become widely used for genetic diagnoses with an excellent cost–benefit ratio in rare disorders and in patients with atypical/challenging phenotypes [9,10,11,12,13]. A single-center exploratory cost-effectiveness analysis of WES in neurogenetic diseases has shown a reduction in the total costs of up to 60% when applying WES compared to a battery of complementary tests performed before WES was available [11]. WES, and more recently, whole-genome sequencing (WGS), have shown substantial diagnostic yields, substantially increasing the number of genetic diagnoses that may be amenable to disease-specific medical management, including, but not limited to, certain types of epileptic encephalopathies and leukoencephalopathies [3,11,14,15]. However, a major challenge is still represented by the interpretation of the pathogenicity of variants not previously reported, and thus, we considered variants of uncertain significance (VUS) [2]. Another important challenge is the discussion of genetic test results (including incidental findings) with the patients [16]. Unfortunately, clinicians have differing opinions on best practices for genetic counseling [16,17].

In 2017, we launched the “Genetics of Rare Movement Disorders” study targeting the evaluation of selected patients with undiagnosed movement disorders of likely genetic etiology (e.g., patients with challenging presentations where a genetic etiology was presumed by the attending physician) in the Gardner Family Center for Parkinson’s Disease and Movement Disorders at the University of Cincinnati (IRB# 2017-5985). The main aims of this study were to determine the extent of phenotypic variability associated with each specific genetic variant and to assess the prevalence of specific genetic variants in our cohort of patients with rare movement disorders (RMDs). The ultimate goal of this project is to influence diagnostic and therapeutic approaches, including the development of early diagnosis and possible novel treatment modalities for several genetically determined movement disorders, and to store the DNA extracted from participants and raw sequencing data in order to be able to periodically re-analyze VUS results or re-examine any undetermined diagnoses, thus improving the diagnostic yield over time, as already outlined in our previous work [3].

In the present manuscript, we aim to describe the results of our RMD study in Cincinnati seven years after its launch and to use this experience as the foundation of a modern movement disorder genetic clinic based on a wide variety of possible genetic tests (e.g., commercially available genetic panels or research-based) to be used according to the individual clinical phenotype and pattern of inheritance.

## 2. Material and Methods

### 2.1. Participants, Locations, and Clinical Data

Patients enrolled in the RMD are adults studied by means of a comprehensive phenotype-guided analysis that includes the DNA sequencing of samples collected from biological materials (blood or saliva). Once the biological material is collected at the University of Cincinnati, DNA is extracted and then stored in different aliquots at the Discover Together Biobank at the Cincinnati Children’s Hospital Medical Center (CCHMC, Cincinnati, OH, USA). DNA sequencing takes place at the CCHMC Genomics Sequencing Facility. The analysis and interpretation of data is then performed at the Neurogenetics Clinic and Laboratory, Neurology Division of the Hospital JM Ramos Mejia in Buenos Aires, Argentina (where the consultant neurogenetics specialist—MAK—is based). Positive results can be confirmed in any clinical laboratory (CLIA) upon the patient’s request. The latter may be required for insurance or healthcare purposes.

The RMD study includes all patients with undiagnosed movement disorders that might potentially benefit from Genomic Analysis without restrictions on gender, ancestry, religion, or social class. Subjects were enrolled after a thorough clinical evaluation at the Gardner Family Center, University of Cincinnati, starting from 30 August 2017–1 April 2024. Moreover, starting from January 2022, subjects were also enrolled at the Universidad de Chile, Santiago, Chile. Subjects are considered for inclusion in the study for comprehensive genetic testing based on the phenotype, specifically a mixed movement disorder phenotype or phenotype that included symptoms of a known/suspect genetic disorder (as outlined in the Supplementary Material File S1 Questionnaire administered to all patients). No specific exclusion criteria apply, except for the age at enrollment, which needed to be ≥18 years old (patients can have any age at onset), given our center’s focus on adult movement disorders. Subjects are seen only once during the study, given its observational and cross-sectional design. After written informed consent is obtained, blood or saliva samples are collected to perform WES. When considered useful, the investigations compare the patient’s genetic findings with those of other family members to understand which genetic variants are associated with the patient’s current disease. The investigators used health information and family health history to help identify the isolated genetic variants likely to be responsible for the patient’s signs and symptoms. For each patient, we collect perinatal, personal, and family history (including family tree, likely pattern of inheritance, disease progression characteristics, comorbidities, and studies performed before WES, using a standardized questionnaire created ad hoc for the study (Supplementary Material File S1 Questionnaire). Other relevant data are collected from the clinical notes of the patient. A detailed videotaped neurological examination is also performed during the visits. The informed consent includes the option to receive incidental findings according to the American College of Medical Genetics (ACMG) recommendations [18]. All methods are performed in accordance with the relevant guidelines and regulations. Results are then discussed with patients and family members, and counseling can be performed by trained professionals on a case-by-case basis according to the need.

The study was approved by the local Ethics Committee of each participating country. All participants signed the written informed consent before participating in the study.

### 2.2. Technical Procedure

The Genomic Analysis test is phenotype-driven and consists of WES evaluating for germinal single nucleotide genetic variants and small exonic duplications or deletions. This test requires a small sample of blood (two 6 mL tubes in total) or saliva (one tube, 2 mL) from the participants. Blood is usually recommended, but saliva can be used if patients prefer. The lab isolates DNA from the sample(s). When necessary, the patient’s genetic findings are compared with those of other family members to understand which genetic variants are associated with the patient’s disease. We use health information and family health history to help identify the genetic variants likely to be responsible for the patient’s signs and symptoms. The Neurogenetics Clinic and Laboratory of Ramos Mejia Hospital (Buenos Aires, Argentina) provides a research report of the Genomic Analysis results to the study team.

### 2.3. Whole Exome Sequencing

Genomic DNA is isolated and extracted on Autogen FlexStar+ automated DNA extractors (Autogen, Boston, MA, USA) following the manufacturer’s instructions and kept anonymous. DNA sequencing libraries are constructed with enzymatic fragmentation, and the exonic regions are enriched using probes from Twist Bioscience (San Francisco, CA, USA). NGS sequencing runs are made in Illumina systems (Illumina, INC). The detection of variants is possible, with an average sequence coverage of more than 90X and with more than 97% of the target bases having at least 60X coverage. All standardized procedures are performed according to the manufacturer’s instructions, which are widely described in the literature [19,20].

### 2.4. Data Analysis

FASTQ files (a text-based format for storing both a nucleotide sequence and its corresponding quality scores) of each subject are aligned to reference the human genome (GRCh37 or GRCh38) using the Burrows–Wheeler Alignment Tool 3 (Illumina, San Diego, CA, USA) [21]. Variant calls are generated using the Genome Analysis Toolkit (GATK) (Broad Institute, Cambridge, MA, USA) haplotype caller (versions 3.6 or 4.1), following the best practices [22]. The output Variant Call Format (VCF) files are annotated at various levels using ANNOVAR (2017 and following versions) (a software tool of up-to-date information to functionally annotate genetic variants detected from diverse genomes) [23], with information from several databases, as previously described [11,12]. Using a pipeline developed by the investigators, all of the variants identified in genes recognized as causing the different movement disorders are classified according to the ACMG recommendations in order to identify subjects that could be considered as suffering from these rare conditions [11,24].

For the vast majority of exomes, virtual multigenic panels are built from a search of genes reported as pathogenic. These standardized virtual panels are central in the clinical interpretation of annotated VCF files. In brief, variants are further prioritized based on the inheritance model for each case, population frequency, predicted molecular function and effects, and previous reports of pathogenicity in other patients with similar clinical diagnosis, using procedures and bioinformatics pathways developed elsewhere [11]. Joining the variant level and clinical features information, each study is then classified as positive, VUS, or negative, as follows:

- Positive: A pathogenic/likely pathogenic variant in a known disease gene is identified with a compatible phenotypic and inheritance overlap.
- VUS: A pathogenic/likely pathogenic variant in a putative candidate gene is identified without positive phenotypic and inheritance overlap; one pathogenic/likely pathogenic variant is identified with a positive phenotypic overlap in a recessive disorder (unable to be detected in the other allele) and one pathogenic/likely pathogenic variants are identified in a potential candidate gene not yet associated with disease; VUS are then further divided into two main categories, according to their odds of pathogenicity [25]: (1) non-diagnostic but with a strong gene candidate (strong-gene VUS candidate) (e.g., a VUS variant with incomplete characteristics to be classified as pathogenic or likely pathogenic, but interpreted with high probability as disease-causing by the genetic analyst—MAK) [25], and (2) non-diagnostic and non-strong candidate (non-strong gene VUS candidate).
- Negative: No variants are identified.

Finally, secondary findings are not reported.

### 2.5. Expansion of Disease-Related Annotations in Biomedical Literature

Data from the Human Genome Mutation Database (HGMD), the OMIM database, MEDLINE, life science journals, and online books are used to quantify the growth in variant–disease, gene–disease, and biomedical literature–neurogenetics disease associations. The number of OMIM gene–disease and HGMD variant–disease associations were established, as previously described [26]. A systematic search is then applied using all clinical diagnoses incorporated in the patient series as keywords through PubMed (National Library of Medicine) to count the biomedical literature–neurogenetics disease associations.

### 2.6. Statistical Analysis

Descriptive statistics, including the mean, standard deviation, counts, and percentages, were used to summarize demographic and clinical characteristics in all subjects and in each group of interest (pathogenic/likely pathogenic, VUS, and negative). For qualitative characteristics, Fisher’s exact test was used. ANOVA and Kruskal–Wallis’ tests were used to assess statistical differences in normally and non-normally distributed quantitative characteristics between the three groups. When the Kruskal–Wallis test indicated differences among the three groups, Dunn’s test with Benjamini–Yekutieli adjustment was performed as a post hoc analysis for multiple pairwise comparisons. The Benjamini–Yekutieli adjustment was employed for Dunn’s test, where false-discovery-rate-adjusted *p*-values were adjusted sequentially by ordering the *p*-values of each pairwise test from the largest to the smallest. Given the exploratory nature of our analysis, only Dunn’s post hoc test—not Fisher’s exact test, ANOVA, or Kruskal–Wallis test—was adjusted for multiple comparisons. Lastly, for the same reason, no sample size calculation was conducted, and all available patients were included in the analysis. In all, a difference was considered statistically significant if the *p*-value was less than 0.05. STATA v.18.0 (StataCorp LP, College Station, TX, USA) was used.

## 3. Results

To date, we have obtained WES in a total of 88 patients. The characteristics of our studied population (and related family IDs) are in Table 1. Of the 88 participants, 55 (63%) were female, 82 (93%) were of European ancestry, and 87 (99%) were of non-Hispanic ethnicity.

There was no difference in terms of sex, ancestry, or ethnicity among patients with pathogenic/likely pathogenic results, VUS, and those without potentially diagnostic variants identified (negative for mutations). The median age at onset was significantly lower in patients with pathogenic/likely pathogenic variants [27.5 years old] when compared to VUS 61 (47–63.5), or negative results [57 (40–65) years old] (both *p*-values < 0.05). The median age at examination was lower in patients with pathogenic/likely pathogenic variants [52 (37–66) years old] when compared to VUS [67 (60–75) years old] or negative results [65.5 (49–71)], but was not statistically significant (*p* = 0.09). The prevalence of a positive family history, namely the presence of at least one family member affected and with a similar phenotype, did not differ between groups (*p* = 0.11) (Table 2).

**Figure 1:**
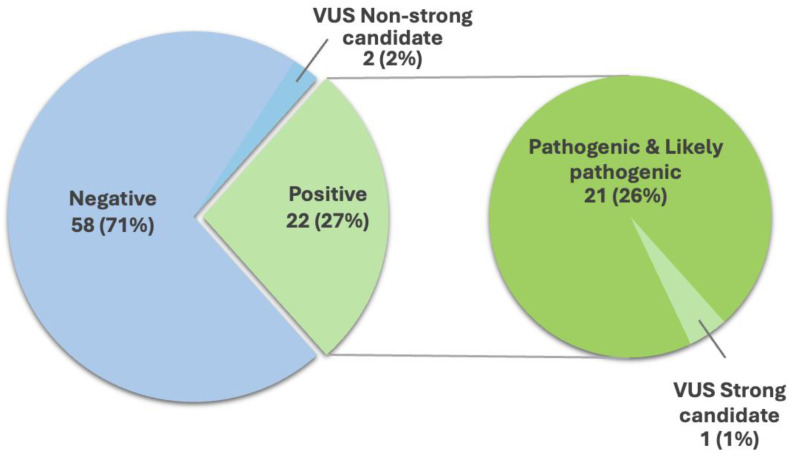
Main results of the study. Green: Positive results (diagnostic yield) reached 27% (22 out of 82 probands) (**left**), of whom 26% (21) with pathogenic or likely pathogenic variants, and 1% (1) with strong-gene VUS candidate (green filled circle) (**right**). Blue: differently, 58% (71) of patients resulted negative, and 2.4% patients (2) resulted as VUS-non strong candidates (**left**).

After excluding six family members from four index participants, the diagnostic yield reached 27% (22 out of 82 probands), 26% (21) with pathogenic or likely pathogenic variants, and 1% (1) with a VUS-strong gene candidate, whereas 58% (71) of patients had negative results, and 2.4% patients (2) were VUS-non strong candidates (Supplementary Table S1 and Figure 1). Twenty-three percent (5 out of 21 probands) of patients diagnosed with pathogenic or likely pathogenic variants were initially considered VUS and then reclassified as pathogenic or likely pathogenic for the following isolated genes: *COL22A1* and *TGM6* based on functional studies [27,28], CP on computational analyses [29], and *LRRK2* on newly confirmed pathogenicity in the literature.

Overall, the most common included phenotypes were ataxia (34 patients), parkinsonism (21 patients), dystonia (with and without spastic syndrome, 7 patients), isolated spastic syndrome (6 patients), chorea (with and without dementia; 6 patients), and leukoencephalopathy (with and without ataxia; 5 patients), with lesser representation of myoclonus, epilepsy, and tremors. The frequency of pathogenic/likely pathogenic mutations was 50% in dystonia (3/6), spastic syndrome (3/6), and epilepsy (1/2); 29% in ataxia (10/34); 25% in leukoencephalopathy (1/4); 24% in parkinsonism (5/21); 20% in chorea (1/5). The isolated genes, their zygosity, and variant types are reported in Supplementary Table S2.

**Figure 2:**
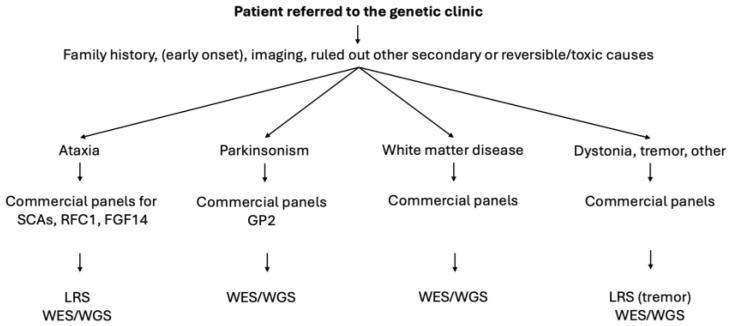
Flowchart of the ideal diagnostic algorithm for patients presenting in the movement disorders genetic clinic. Based on the family history, brain imaging, and clinical phenotype; after having ruled out the most common and reversible causes, patients undergo genetic testing. First, commercial panels or sequencing of single genes is recommended, based on costs and patients’ availabilities. Then, if negative or of uncertain significance, testing through research avenues is recommended. SCAs, spinocerebellar ataxia; RFC1, replication factor C subunit 1—gene of cerebellar ataxia, neuropathy and vestibular areflexia—CANVAS; FGF14, fibroblast growth factor 14—gene of SCA27B; GP2, Global Parkinson’s Genetics Program; WES, whole-exome sequencing; WGS, whole-genome sequencing.

Based on these results, we have developed a protocol to prioritize genetic testing for clinical purposes, considering both the cost and likelihood of genetic diagnosis. We offer an initial genetic panel commercially available for parkinsonism, ataxia (including repeat-expansion disorders), dystonia, and leukoencephalopathies, depending on the clinical phenotype, and with low costs for patients (not often covered by insurance). If the results are negative/inconclusive, we move forward with more detailed genetic analyses, often performed under research coverage but with the caveat that any identified variants would need to be verified in a clinical laboratory (CLIA). The second-level genetic testing is WES through the RMD study or long-read sequencing (LRS) in the case of suspected repeat-expansion disorders (Figure 2).

## 4. Discussion

We describe the experience of a seven-year-duration project in Cincinnati designed to offer WES-based genetic testing to patients with undiagnosed movement disorders. Out of the 82 individual probands studied, 26% (21) have been diagnosed with genetic variants deemed responsible for their conditions, and 1% (1) with VUS-strong gene candidates. The age at onset was significantly lower in patients with pathogenic/likely pathogenic variants, and the most common clinical phenotypes were ataxia and parkinsonism. Overall, dystonia (with and without spastic syndrome), ataxia, leukoencephalopathy, and parkinsonism were the clinical categories with the most frequent genetic diagnoses.

The diagnostic yield obtained using WES in our previous study was reported to be around 20–25% [3]; hence, we showed an increased success over time if we considered VUS-strong gene candidates as part of the diagnostic success. Other studies have reported variable ranges of success, ranging from 25% [14] to 40% [11], but in different neurologic conditions (including epileptogenic conditions, with a clear pattern of inheritance) and with heterogeneous ages of participants (including the pediatric populations where these conditions tend to be more frequent). Patients with pathogenic/likely variants, as expected in many genetic disorders, tend to have an earlier age at onset and present mostly with dystonia (even with spasticity), ataxia, and parkinsonism (with and without leukoencephalopathy) in an adult movement disorders clinic [30].

Three (3.4%) of the studied patients were from Chile as part of our collaboration project launched in 2022. In line with the idea of inclusivity and equity, this project was created to offer sophisticated genetic analyses for non-European populations, especially those with a minority ethnic background. The reason behind this is that there is a lack of diversity in genetic research, with most genetic studies recruiting mostly people of European ancestry. We aim to increase the number of participants from Chile to be studied over time, to allow this population to be more represented, and to offer high-level genetic testing otherwise unavailable through commercial panels in non-US and non-European populations.

Regarding positive findings, in some cases, these results helped with the counseling of probands and their related family members in obtaining an answer on the causes of their diseases, their related transmission over the generations (AD vs. AR, vs. X-linked), and the risk of disease development for the new/upcoming generations. In five cases, the isolated variants also allowed prioritization of the drugs to be used in order to alleviate symptoms (in the three patients with *CACNA1A* point mutations, acetazolamide followed by verapamil, valproic acid, and gabapentin were used; in *KNCH1* channelopathy benzodiazepines and levetiracetam were tried; and in *SCN4A* mutation a sodium channel locker like mexiletine has been offered). Also, regarding the issue of VUS, we found that in 62.5% (5/8) of cases, the accurate investigation of these variants through functional studies (drosophila or cell-based), computational analyses (protein modeling studies), and reassessment of the mutations over time in the available databases allowed us to prove their deleterious function and clarify the role of the identified variants, thus transitioning from strong VUS candidates to pathogenic/likely pathogenic variants [27,28,29]. We discussed each single case among neurology providers and geneticists to identify the VUS cases suitable for functional assays, even if this was beyond the scope of our primary research objectives. Then, we identified selected laboratories capable of helping with functional assays for these strong VUS candidates (using research funding).

After a seven-year-history of this collaborative effort, we would like to offer some reflections on the design of a modern genetic clinic and outline a diagnostic algorithm for the prioritization of genetic testing to obtain the most appropriate and highest-quality analyses with the lowest cost to our patients (when commercially performed) and institution (when performed for research purposes) (Figure 2). The present protocol has several strengths. First, one of the intrinsic limitations of routine NGS techniques is their scarce ability to decipher structural DNA variations (e.g., structural variants or copy number variants, which include tri-, tetra-, and penta-nucleotide repeat expansion disorders) [15], given their ability to only sequence short-read length fragments [3]. Technological developments have recently allowed for increasing the length of sequencing reads, giving space to this new technology named LRS, with the added potential to study repeat expansions in patients with ataxia, familial tremor disorders, or even leukoencephalopathies [15]. The LRS technique is included in our protocol (LRS analysis has not been completed due to costs), thus allowing the diagnosis of repeat-expansion disorders. However, in our study protocol, we do not include patients with Huntington’s disease (HD), given that all cases with suspect HD are referred to our Center of Excellence for HD and receive commercial testing billed through insurance. Additionally, in the case of adult-onset leukoencephalopathies, we have an ongoing project with WGS analysis that allows extensive testing but with elevated research costs. Finally, we are a collaborative center of the Global Parkinson’s Genetics Program (GP2) study [31], and we refer all patients with suspect monogenic parkinsonism to the “Monogenic Hub”, thus receiving WGS analyses for these selected cases. This is a proposed algorithm based on availabilities at our center. Each institution should work with its geneticists and partner labs to obtain the most suitable/accurate testing available for its patients, considering all direct and indirect related costs.

Since the launch of the human genome project in 1990 (total duration 13 years, total cost of about five billion USD) [32], the duration and costs for a thorough analysis of human DNA have dropped significantly; however, there are still associated substantial direct and indirect costs for a WES analysis when performed for research in an academic institution. We have estimated that this amount ranges between $800 and $1000 USD per patient for WES analysis and includes DNA extraction, storage (for DNA banking, with additional prices over the years), sequencing, and interpretation, with some variability related to the level of coverage and intrinsic quality of analysis (but does not include retesting over time). For WGS, the price at least doubles.

The more affordable costs for thorough genetic analyses have enabled the expansion of the disease spectrum of many genetic disorders [15] but led to several unexpected or secondary findings as well as to the detection of VUS with undetermined consequences. Hence, the delivery of genetic information to patients and their family members (possible carriers in some situations) requires dedicated pre- and post-testing genetic counseling [33]. The available genetic counseling services vary from center to center and from country to country, with several related ethical and legal issues. In our center, we have a dedicated movement disorder clinical geneticist (currently made possible through philanthropic donations), and we also rely on other possible resources, such as those offered by selected studies for their participants [32]. The issue of return of research results is also a currently debated topic in the GP2 study for Parkinson’s disease patients, with multiple implications that may vary according to the country or region and do not have a clear or immediate solution [16]. In any case, it is important to keep patients informed on all of the potential challenges before signing the consent form for study enrollment. In our genetic clinic, in partnership with the CCHMC, we will start using a document as part of the consent form that clearly explains what WES is and what primary and secondary findings are and includes an opportunity to opt-out from learning the results of secondary findings. This will include all the related health, emotional, and insurance considerations [34]. It is important to underline that the emphasis on the disclosure of results is for those with confirmed clinical utility where there is the possibility of treatment/prevention, not other incidental findings of unclear relevance or a relationship to the phenotype. In genetic clinics, clinicians should also be able to provide genetic counseling to symptomatic patients. The education of neurologists on complex genotype–phenotype correlations is important to ensure they are confident in explaining the genetic results. In a future world with widespread genetic testing, it is important to acknowledge that it will not always be feasible to have a genetic counselor for everyone, and the repercussions of such a shortcoming will require discussion.

A final comment is related to the possibility of repeating genetic analysis over time to increase the diagnostic yield. In fact, the RMD study stores the DNA in our BioBank, as well as the raw WES files in dedicated cloud platforms, thus having the possibility to repeat genetic analyses over time; for instance, in patients with VUS or with negative results but with suspected genetic conditions (e.g., strong family history with specific pattern of inheritance, peculiar clinical phenotype) who may benefit from a WGS after a WES analysis. As documented in a previous re-analysis of stored raw sequencing data up to 2020, the diagnostic yield may increase from 20% to 26% while adopting this approach [3]; hence, given our current diagnostic yield of 27%, we expect to increase it further. Another possible explanation for negative WES results may come from the further clarification of the role of VUS through in vitro or in vivo animal studies as well as protein modeling computational analyses able to clarify possible concurrent deleterious effects of multiple variants (pathogenic, likely-pathogenic, and VUS) on protein functions [29].

The present study has some limitations, including the sample size due to the rarity of the diseases and the lack of a longitudinal follow-up approach. We included any age at onset to avoid imposing limitations or barriers, but this likely had an impact on the diagnostic yield of genetic testing; in fact, younger age at dystonia onset has been associated with an increased diagnostic yield [8]. However, in our cohort, all but nine patients had an age at onset greater or equal to 18 years old, and only two patients out of these nine had dystonia as a symptom. Also, 93% of our patients were of European ancestry, thus limiting the generalizability of our results, and patients with ataxia are more likely to have repeat expansion disorders, which are not captured in most WES. This technical limitation is precisely why we developed our tiered diagnostic approach (Figure 2), where WES is part of a comprehensive genetic testing strategy rather than a standalone test. The complementary use of targeted panels for repeat expansions, WES for point mutations and small indels, and LRS for complex structural variants allows us to maximize our diagnostic yield while being cost-effective. This approach is particularly important given that our cohort included numerous patients with ataxia and other conditions commonly associated with repeat expansions. This multi-modal approach allows us to leverage the advantages of WES (cost-effectiveness, ability to detect novel variants, good coverage of coding regions) while acknowledging and addressing its limitations through complementary testing strategies when clinically indicated. Finally, we are aware that, in this study, the subjective nature of case selection may have introduced biases, and the lack of predefined sample size calculation likely affected the capacity to identify statistically significant differences between particular groups. We aim to develop a more systematic approach to case selection in the future.

## 5. Conclusions

In conclusion, based on our findings, we now aim to implement the protocol for our genetic clinic. This includes a series of decision trees for when to consider genetic testing, how to prioritize which genetic test should be undertaken, how and when to further investigate strong VUS candidate results through functional assays, how best to return results, and how to offer/monitor response to treatment options tailored to the genetic result. The ultimate goal is not merely to achieve a genetic diagnosis but rather to consider it as a starting point to (a) Prioritize conditions suitable to be tested in disease-modifying trials already available for monogenic disorders [4,5]; (b) Create databases and networks connecting experts on rare genetic conditions with the goal of increasing knowledge of their clinical presentation and management as well as to be ready to enroll patients once related trials become available (e.g., through the GP2 study); (c) Test new diagnostic and pathophysiological hypotheses in the field of neurodegeneration, where monogenic conditions may explain, at least in part, some of these neurodegenerative mechanisms [33], also considering the role of multi-omic data integration, which might optimize diagnostic success by combining genomic, epigenetic, transcriptomic, and proteomic information to allow a broader evaluation of variant effects [35].

## Figures and Tables

**Table 1:** Overall participants’ characteristics.

Characteristics	Overall, N = 88
**Sex**	
F	
(%)	55 (63%)
M	
(%)	33 (38%)
**Ancestry**	
African American	
(%)	5 (5.72%)
European	
(%)	82 (93.2%)
Other	
(%)	1 (1.08%)
**Ethnicity**	
Hispanic or Latino	1 (1.1%)
**Excluded** (Family members of probands)	6 (6.8%)

Data is presented as *n* (%), or mean (standard deviation—SD), as appropriate. F, female, M, male.

**Table 2:** Demographic differences in participants between pathogenic/likely pathogenic, variants of unknown significance, and negative results.

Characteristics	Pathogenic/ Likely, N = 26	VUS, N = 4	Negative, N = 58	*p*-Value	Pathogenic/Likely vs. VUS	Pathogenic/Likely vs. no Mutation	VUS vs. Negative
**Sex**				0.14			
F (%)	19 (73%)	1 (25%)	35 (60%)				
M (%)	7 (27%)	3 (75%)	23 (40%)				
**Ancestry**				0.8			
African American (%)	2 (8%)	0 (0%)	3 (5%)				
European (%)	22 (92%)	7 (100%)	53 (93%)				
Hispanic (%)	0 (0%)	0 (0%)	1 (1.8%)				
				>0.99			
Hispanic or Latino (%)	0 (0%)	0 (0%)	1 (2%)				
Non-Hispanic (%)	26 (100%)	4 (100%)	57 (98%)				
**Age at symptoms onset**	27.5 5	61 (47–63.5)	57 (40–65)	0.02	0.24	0.02	0.78
**Age at examination**	52 (37–66)	67 (60–75)	65.5 (49–71)	0.09			
**Number of relatives**	1.4 (1.8)	1.5 (1.3)	0.7 (1.3)	0.11			

Data is presented as median (percentile 25–percentile 75), mean (standard deviation) or *n* (%), as appropriate. VUS, variant of unknown significance, F, female, M, male.

## Data Availability

Any anonymized data not published within the article can be shared by request from any qualified investigator due to Privacy.

## Supplementary Materials

The following supporting information can be downloaded at https://www.mdpi.com/article/10.3390/biomedicines12122673/s1, File S1: Questionnaire. Screening questionnaire administered to all study participants; Table S1: Pathogenic, likely pathogenic, and variant of unknown significance identified in the study; Table S2: Patients’ phenotypes and isolated genes. Reference [36] is cited in the Supplementary Materials.
