# Supplementary material for: Studying Rare Movement Disorders: From Whole-Exome Sequencing to New Diagnostic and Therapeutic Approaches in a Modern Genetic Clinic
Source: Biomedicines. 2024 Nov 23;12(12):2673. doi: 10.3390/biomedicines12122673 (PMC11727247; doi:10.3390/biomedicines12122673)
Supplement: Supplementary file 1 [file biomedicines-12-02673-s001.zip › Supplementary Questionnaire.pdf]

SCREENING QUESTIONNAIRE

Genetics of Unusual and Familial Movement Disorders: Clinical epidemiology and genetic study

1. **DEMOGRAPHIC (for patient)**

Case Report Form [CRF]

FIRST NAME: …………………………………

LAST NAME: …………………………………

DATE OF BIRTH (mm/dd/yyyy): ………………………………………………

AGE (years): ………………………………………………

PATIENT ID: …………………………………

DATE of the Examination: ……………………………………………………………………

- GENDER: ☐ **MALE** ☐ **FEMALE**

- RACE:

☐ **Caucasian**

☐ **African American**

☐ **Asian**

☐ **American Indian / Alaska native**

☐ **Native Hawaiian / other Pacific Islander**

☐ **Unknown**

☐ **Other (Specify: ……………………………………………….)**

- ETHNICITY:

☐ **Hispanic or Latino**

☐ **Not Hispanic or Latino**

☐ **Ashkenazi Jewish**

☐ **Mennonite**

☐ **Unknown**

☐ **Other (Specify: ……………………………………………………)**

- ADDITIONAL INFORMATION, IF KNOWN:

Is there any of the following background in your family?

From Southeast Asia, Taiwan, China, or the Philippines? ☐ **YES** ☐ **NO** ☐ **UNKNOWN**

If yes, please specify: ………………………………………………………………………

From Italy, Greece (Mediterranean), or the Middle East? ☐ **YES** ☐ **NO** ☐ **UNKNOWN**

If yes, please specify: ………………………………………………………………………

From Africa or African-American? ☐ **YES** ☐ **NO** ☐ **UNKNOWN**

If yes, please specify: ………………………………………………………………………

Central Eastern European? ☐ **YES** ☐ **NO** ☐ **UNKNOWN**

If yes, please specify: ………………………………………………………………………

Cajun or French Canadian? ☐ **YES** ☐ **NO** ☐ **UNKNOWN**

If yes, please specify: ………………………………………………………………………

- HEIGHT:

…………. (In Feet) or …………. (In Centimeters)

- WEIGHT:

…………. (In Pounds) or …………. (In Kilograms)

- AGE AT ONSET (years): ………………………………………………

- HOW MANY CHILDREN DO YOU HAVE? …………………………………….

- ONLY FOR WOMEN:

GRAVIDA (Total number of pregnancies): …………………………………….

PARA (Total number of live births): …………………………………….

MISCARRIAGE(S) (If yes, specify the total number): …………………………………….

MENSTRUAL HISTORY:

AGE AT FIRST PERIOD …………………………………….

ONLY FOR PATIENTS IN MENOPAUSE:

AGE AT MENOPAUSE …………………………………….

- TIME SINCE SYMPTOM ONSET (In years): ………………………………………………

- TIME SINCE DIAGNOSIS (In years): ………………………………………………

- RELATIVE(S) WITH NEUROLOGICAL DISORDERS? ☐ **YES** ☐ **NO** ☐ **UNKNOWN**

- FIRST DEGREE FAMILY HISTORY OF NEUROLOGICAL DISORDERS?

☐ **YES** ☐ **NO** ☐ **UNKNOWN**

If Yes, please specify…………………………………………………….

……………………………………………………………………………

……………………………………………………………………………

- FAMILY HISTORY OF ANY NEUROLOGICAL DISORDERS? ☐ **YES** ☐ **NO** ☐ **UNKNOWN**

If Yes, please, specify…………………………………………………….

If Yes, specify disease, family degree (2nd, 3rd) and paternal versus maternal line:

……………………………………………………………………………………..

……………………………………………………………………………………..

…………………………………………………………………………………………………..

- FAMILY HISTORY OF ANY OTHER DISORDER? ☐ **YES** ☐ **NO** ☐ **UNKNOWN**

If Yes, please specify……………………………………………………….

……………………………………………………………………………

……………………………………………………………………………

- In particular, have any member of your father's/mother's family ever been diagnosed any of the following disorders?

Down syndrome ☐ **YES** ☐ **NO** ☐ **UNKNOWN**

Other chromosomal abnormalities ☐ **YES** ☐ **NO** ☐ **UNKNOWN**

Neural tube defect (spina bifida) ☐ **YES** ☐ **NO** ☐ **UNKNOWN**

Bleeding disorder (hemophilia) ☐ **YES** ☐ **NO** ☐ **UNKNOWN**

Cystic fibrosis ☐ **YES** ☐ **NO** ☐ **UNKNOWN**

Sickle Cell ☐ **YES** ☐ **NO** ☐ **UNKNOWN**

Thalassemia ☐ **YES** ☐ **NO** ☐ **UNKNOWN**

Tay-Sachs/Canavan ☐ **YES** ☐ **NO** ☐ **UNKNOWN**

Neurofibromatosis ☐ **YES** ☐ **NO** ☐ **UNKNOWN**

Bone or skeletal disorder ☐ **YES** ☐ **NO** ☐ **UNKNOWN**

Polycystic kidney disease ☐ **YES** ☐ **NO** ☐ **UNKNOWN**

Heart defect (at birth) ☐ **YES** ☐ **NO** ☐ **UNKNOWN**

Cleft lip/palate ☐ **YES** ☐ **NO** ☐ **UNKNOWN**

- Were your parents (or grandparents) related by blood; for example, cousins?

☐ **YES** ☐ **NO** ☐ **UNKNOWN**

If Yes, please, specify**……………………………………………………….**

- Was there any member of your family with a genetic condition or chromosomal abnormality not listed above?

☐ **YES** ☐ **NO** ☐ **UNKNOWN**

If Yes, please, specify**……………………………………………………….**

- Was there any member of your family with a birth defect no listed above?

☐ **YES** ☐ **NO** ☐ **UNKNOWN**

If Yes, please, specify**……………………………………………………….**

- PROFESSION (if retired, clarify prior occupation): **………………………………………………**

- For how long? **………………………………………………**

- SMOKER: ☐ **YES** ☐ **NO** ☐ **FORMER SMOKER**

- If yes, number of packs/day: **…………………………………**

- If former, number of packs/day and number of years of smoking until quitting: **……………..**

- ALCOHOL: ☐ **YES** ☐ **NO** ☐ **OCCASIONALLY** ☐ **FORMER DRINKER**

- If yes, number of drinks/day: **…………………………………**

- If former, number of glasses or bottles per day and years of drinking: **……………..**

- CAFFEINE: ☐ **YES** ☐ **NO** ☐ **OCCASIONALLY** ☐ **FORMER DRINKER**

- If yes, number of coffee/day: **…………………………………**

- If former, number of cups/day and years of drinking: **……**

2. CLINICAL (for the clinician)

-Draw here a family tree:

-Summary of the case (100-150 words):

…………………………………………………………………………..……………….

…………………………………………………………………………..……………….

…………………………………………………………………………..……………….

…………………………………………………………………………..……………….

…………………………………………………………………………..……………….

Cognitive/Behavioral Function

Autism Spectrum Disorders ☐ **YES** ☐ **NO** ☐ **UNKNOWN**

Depression ☐ **YES** ☐ **NO** ☐ **UNKNOWN**

Auditory hallucinations ☐ **YES** ☐ **NO** ☐ **UNKNOWN**

Visual hallucinations ☐ **YES** ☐ **NO** ☐ **UNKNOWN**

Behavioral problems ☐ **YES** ☐ **NO** ☐ **UNKNOWN**

Intellectual problems ☐ **YES** ☐ **NO** ☐ **UNKNOWN**

Dementia ☐ **YES** ☐ **NO** ☐ **UNKNOWN**

Mild cognitive impairment ☐ **YES** ☐ **NO** ☐ **UNKNOWN**

Speech disturbances or Aphasia ☐ **YES** ☐ **NO** ☐ **UNKNOWN**

Speech disturbances or Dysarthria ☐ **YES** ☐ **NO** ☐ **UNKNOWN**

Neurological Symptoms

Cranial nerves

Optic Neuropathy ☐ **YES** ☐ **NO** ☐ **UNKNOWN**

Ophthalmoparesis/ptosis ☐ **YES** ☐ **NO** ☐ **UNKNOWN**

Vertical gaze palsy ☐ **YES** ☐ **NO** ☐ **UNKNOWN**

Nystagmus ☐ **YES** ☐ **NO** ☐ **UNKNOWN**

Square-Wave Jerks ☐ **YES** ☐ **NO** ☐ **UNKNOWN**

Slowness of saccades ☐ **YES** ☐ **NO** ☐ **UNKNOWN**

Peripheral facial palsy ☐ **YES** ☐ **NO** ☐ **UNKNOWN**

Central facial palsy ☐ **YES** ☐ **NO** ☐ **UNKNOWN**

Dysphagia ☐ **YES** ☐ **NO** ☐ **UNKNOWN**

Hypoacusis ☐ **YES** ☐ **NO** ☐ **UNKNOWN**

<u>Cerebellar dysfunction/Ataxia</u>

Truncal ataxia ☐ **YES** ☐ **NO** ☐ **UNKNOWN**

Limb ataxia ☐ **YES** ☐ **NO** ☐ **UNKNOWN**

Sensory ataxia ☐ **YES** ☐ **NO** ☐ **UNKNOWN**

<u>Movement disorders</u>

Parkinsonism ☐ **YES** ☐ **NO** ☐ **UNKNOWN**

Tremor ☐ **YES** ☐ **NO** ☐ **UNKNOWN**

Tics ☐ **YES** ☐ **NO** ☐ **UNKNOWN**

Chorea ☐ **YES** ☐ **NO** ☐ **UNKNOWN**

Athetosis ☐ **YES** ☐ **NO** ☐ **UNKNOWN**

Dyskinesia ☐ **YES** ☐ **NO** ☐ **UNKNOWN**

Generalized dystonia ☐ **YES** ☐ **NO** ☐ **UNKNOWN**

Focal dystonia ☐ **YES** ☐ **NO** ☐ **UNKNOWN**

Myoclonus ☐ **YES** ☐ **NO** ☐ **UNKNOWN**

<u>Pyramidal dysfunction</u>

Hyperreflexia ☐ **YES** ☐ **NO** ☐ **UNKNOWN**

Spasticity ☐ **YES** ☐ **NO** ☐ **UNKNOWN**

Hypertonia ☐ **YES** ☐ **NO** ☐ **UNKNOWN**

<u>Myopathy/Neuropathy/Neuronopathy</u>

Muscular weakness ☐ **YES** ☐ **NO** ☐ **UNKNOWN**

Hypotonia ☐ **YES** ☐ **NO** ☐ **UNKNOWN**

Contractures ☐ **YES** ☐ **NO** ☐ **UNKNOWN**

Hyporeflexia/Areflexia ☐ **YES** ☐ **NO** ☐ **UNKNOWN**

Fasciculation ☐ **YES** ☐ **NO** ☐ **UNKNOWN**

Muscular atrophy ☐ **YES** ☐ **NO** ☐ **UNKNOWN**

Myotonia ☐ **YES** ☐ **NO** ☐ **UNKNOWN**

EMG abnormalities ☐ **YES** ☐ **NO** ☐ **UNKNOWN**

(Specify) **………………………………………………..**

<u>Neuroimaging</u>

Cerebellar atrophy ☐ **YES** ☐ **NO** ☐ **UNKNOWN**

Cortical focal atrophy ☐ **YES** ☐ **NO** ☐ **UNKNOWN**

Cortical diffuse atrophy ☐ **YES** ☐ **NO** ☐ **UNKNOWN**

White matter abnormalities ☐ **YES** ☐ **NO** ☐ **UNKNOWN**

Leukodystrophy ☐ **YES** ☐ **NO** ☐ **UNKNOWN**

Hypomyelination ☐ **YES** ☐ **NO** ☐ **UNKNOWN**

Basal ganglia accumulation ☐ **YES** ☐ **NO** ☐ **UNKNOWN**

Basal ganglia hyperintensities ☐ **YES** ☐ **NO** ☐ **UNKNOWN**

Callosum corpus agenesis ☐ **YES** ☐ **NO** ☐ **UNKNOWN**

Lissencephaly ☐ **YES** ☐ **NO** ☐ **UNKNOWN**

Periventricular heterotopy ☐ **YES** ☐ **NO** ☐ **UNKNOWN**

Hemimegalencephaly ☐ **YES** ☐ **NO** ☐ **UNKNOWN**

Cortical focal dysplasia ☐ **YES** ☐ **NO** ☐ **UNKNOWN**

Cerebellar hypoplasia/Joubert syndrome ☐ **YES** ☐ **NO** ☐ **UNKNOWN**

Other (Specify) **……………………………………………………..**

<u>Epilepsy</u>

Febrile seizures ☐ **YES** ☐ **NO** ☐ **UNKNOWN**

Focal epilepsy ☐ **YES** ☐ **NO** ☐ **UNKNOWN**

(Specify) **……………………………………………………..**

Generalized epilepsy ☐ **YES** ☐ **NO** ☐ **UNKNOWN**

(Specify, including age at onset) **……………………………………………………..**

Response to pharmacological treatment ☐ **YES** ☐ **NO** ☐ **UNKNOWN**

Other

Hyperpigmentation ☐ **YES** ☐ **NO** ☐ **UNKNOWN**

Hypopigmentation ☐ **YES** ☐ **NO** ☐ **UNKNOWN**

Cataracts ☐ **YES** ☐ **NO** ☐ **UNKNOWN**

Retinopathy ☐ **YES** ☐ **NO** ☐ **UNKNOWN**

Migraine ☐ **YES** ☐ **NO** ☐ **UNKNOWN**

Dental abnormalities ☐ **YES** ☐ **NO** ☐ **UNKNOWN**

Scoliosis ☐ **YES** ☐ **NO** ☐ **UNKNOWN**

Pes cavus ☐ **YES** ☐ **NO** ☐ **UNKNOWN**

Hyper laxity of ligaments ☐ **YES** ☐ **NO** ☐ **UNKNOWN**

Gonadic abnormalities ☐ **YES** ☐ **NO** ☐ **UNKNOWN**

Endocrinological abnormalities ☐ **YES** ☐ **NO** ☐ **UNKNOWN**

(Specify) **………………………………………………..**

Diabetes ☐ **YES** ☐ **NO** ☐ **UNKNOWN**

Kidney abnormalities ☐ **YES** ☐ **NO** ☐ **UNKNOWN**

(Specify) **………………………………………………..**

Liver disorders ☐ **YES** ☐ **NO** ☐ **UNKNOWN**

Laboratory abnormalities

Organic aciduria ☐ **YES** ☐ **NO** ☐ **UNKNOWN**

Hyperlactacidemia ☐ **YES** ☐ **NO** ☐ **UNKNOWN**

Hypoglycorrhachia ☐ **YES** ☐ **NO** ☐ **UNKNOWN**

Hyperalaninemia ☐ **YES** ☐ **NO** ☐ **UNKNOWN**

Alpha fetoprotein ☐ **YES** ☐ **NO** ☐ **UNKNOWN**

CPK ☐ **YES** ☐ **NO** ☐ **UNKNOWN**

Increased level of pyruvic acid ☐ **YES** ☐ **NO** ☐ **UNKNOWN**

Other ☐ **YES** ☐ **NO** ☐ **UNKNOWN**

(Specify) **………………………………………………..**

Treatment: Do you take any medication? If yes, please specify:

……………………………………………………………………………………..

……………………………………………………………………………………..

……………………………………………………………………………………..

……………………………………………………………………………………..

……………………………………………………………………………………..

Comorbidities

- OTHER MEDICAL CONDITIONS:

…………………………………………………………………………………………..

…………………………………………………………………………………………..

…………………………………………………………………………………………..

- OTHER THERAPIES (please, specify):

…………………………………………………………………………………………..

…………………………………………………………………………………………..

…………………………………………………………………………………………..
