# Supplementary material for: Studying Rare Movement Disorders: From Whole-Exome Sequencing to New Diagnostic and Therapeutic Approaches in a Modern Genetic Clinic
Source: Biomedicines. 2024 Nov 23;12(12):2673. doi: 10.3390/biomedicines12122673 (PMC11727247; doi:10.3390/biomedicines12122673)
Supplement: Supplementary file 1 [file biomedicines-12-02673-s001.zip › Supplementary Table S1.pdf]

Supplementary Table S1.

Case	Sex	Family ID	Main symptom	Age at symptom onset	Age at examination	Zygosity	Gene	Variant interpretation[a] (odds of pathogenicity)[b]	Variant	Variant type	Pathology report	Published case report
1	F	8	ataxia	16	53	hetero.	*CACNA1A*	pathogenic	NM_001127221:exon13:c.1748G>A:p.R583Q	point, missense		[36]
2	F	8	ataxia	30	66	hetero.	*CACNA1A*	pathogenic	NM_001127222.2:exon13:c.1745G>A:p.R582Q	point, missense		
3	M	8	ataxia	22	45	hetero.	*CACNA1A*	pathogenic	NM_001127222.2:exon13:c.1745G>A:p.R582Q	point, missense		
4	F	25	ataxia	20	51	hetero.	*AFG3L2*	pathogenic	NM_006796:exon16:c.1996A>G:p.M666V	point, missense		
5	F	–	ataxia	23	37	compound hetero.	*SYNE1*	likely pathog.	NM_033071:exon77:c.13112dupG:p.Q4372Pfs*31	duplication, frameshift		
								likely pathog.	NM_033071:exon76:c.12392delA:p.K4131Rfs*10	deletion, frameshift		
6	F	–	ataxia	25	45	compound hetero.	*SYNE1*	likely pathog.	NM_033071:exon77:c.14077C>T:p.R4693X	point, nonsense		
								likely pathog.	NM_033071:exon66:c.10488delA:p.K3496Nfs*13	deletion, frameshift		
7	F	–	ataxia	52	59	hetero.	*TGM6*	likely pathog.	NM_198994:exon5:c.616A>C:p.T206P	point, missense	PSP	[27]
8	M	–	ataxia	12	28	hetero.	*KCNH1*	likely pathog.	NM_002238:exon8:c.1458T>G:p.H486Q	point, missense		
9	F	–	ataxia	54	65	hetero.	*OPTN*	likely pathog.	NM_021980:exon11:c.1344G>C:p.K448N	point, missense		
10	M	–	ataxia	42	61	hetero.	*SOD1, CP*	likely pathog.	NM_000454:exon4:c.347G > A:p.R116H	point, missense		[29]
11	F	–	parkinsonism	12	22	homozy.	*PRKN*	pathogenic	NM_004562.3:c.101_102del; p.Gln34fs	deletion, frameshift		
12	M	3	parkinsonism	58	62	hetero.	*LRRK2*	likely pathog.	NM_198578:exon37:c.5385G>T:p.L1795F	point, missense		

13	F	3	parkinsonism	68	68	hetero.	*LRRK2*	likely pathog.	NM_198578:exon37:c.5385G>T:p.L1795F	point, missense		
14	M	–	parkinsonism	72	77	hetero.	*POLG*	likely pathog.	NM_001126131:exon18:c.2902C>T:p.Q968X	point, nonsense		
15	M	–	parkinsonism	34	39	hetero.	*VPS13C*	likely pathog.	NM_020821:exon55:c.7062+1G>A	point, missense, splice donor		
16	F	–	dystonia	62	70	compound hetero.	*COQ2*	pathogenic[c]	NM_015697:exon7:c.1197delT:p.N401Ifs*14	deletion, frameshift		
								US (18.71)	NM_015697:exon3:c.601G>A:p.A201T	point, missense		
17	F	–	dystonia	3	23	hetero.	*SCN4A*	pathogenic	NM_000334:exon22:c.3917G>C:p.G1306A	point, missense		
18	F	13	spasticity	67	75	hetero.	*ATP5MC3*	pathogenic	NM_001002258:exon4:c.318C>G:p.N106K	point, missense		
19	F	13	spasticity	0	48	hetero.	*ATP5MC3*	pathogenic	NM_001002258:exon4:c.318C>G:p.N106K	point, missense		
20	F	13	spasticity	0	24	hetero.	*ATP5MC3*	pathogenic	NM_001689:exon5:c.318C>G:p.N106K	point, missense		
21	F	–	dystonia	5	36	hetero.	*GCH1*	likely pathog.	NM_000161.3:exon6:c.677_678del:p.Val226fs	deletion, frameshift		
22	F	–	chorea	6	26	hetero.	*KCNQ2*	pathogenic	NM_004518:exon4:c.619C>T:p.R207W	point, missense		
23	F	–	epilepsy	8	39	hetero.	*POLG*	pathogenic	NM_001126131:exon2:c.158_159insGCA:p.Q55_P56insQ	insertion, frameshift		
24	F	–	parkinsonism	67	73	hetero.	*COL22A1*	likely pathog.	NM_152888:exon61:c.4268G>A:p.G1423D	point, missense		[28]
25	M	5	parkinsonism	60	81	hetero.	*SLC20A2*	US (9)	NM_001257180:exon6:c.715C>T:p.R239W	point, missense	PD	
26	M	5	parkinsonism	65	69	hetero.	*SLC20A2*	US (9)	NM_001257180:exon6:c.715C>T:p.R239W	point, missense		
27	M	–	parkinsonism	62	65	hetero.	*PDE10A*	US (38.91)[c]	NM_001130690:exon16:c.1531T>G:p.F511V	point, missense		
28	F	–	parkinsonism	34	55	hetero.	*DNAJC13*	US (9)[c]	NM_015268.4:exon40:c.4544C>T:p.Pro1515Leu	point, missense		
29	F	–	ataxia	65	68	hetero.	*SACS*	benign	NM_001278055:exon8:c.7952C>A:p.P2651Q	point, missense		

30	F	–	ataxia	56	60	hetero.	*RRM2B*	likely pathog.[c]	NM_015713:exon6:c.674G>A:p.S225N	point, missense		
31	M	–	myoclonus	86	94	hetero.	*GNAO1*	likely pathog.	NM_020988:exon4:c.446T>A:p.L149H	point, missense		

[a] According to Richards et al., 2015 [22]. For further details, see text.

[b] According to Tavtigian et al., 2018 [23]. For further details, see text.

[c] Interpreted as non-diagnostic but as strong candidate pending further evidence.

Hetero., heterozygous; homozy., homozygous; LKO, leukoencephalopathy; pathog., pathogenic; US, uncertain significance; ID, identification number; M, male; F, female; PSP, progressive supranuclear plasy; PD, Parkinson's disease.
