# Supplementary material for: Studying Rare Movement Disorders: From Whole-Exome Sequencing to New Diagnostic and Therapeutic Approaches in a Modern Genetic Clinic
Source: Biomedicines. 2024 Nov 23;12(12):2673. doi: 10.3390/biomedicines12122673 (PMC11727247; doi:10.3390/biomedicines12122673)
Supplement: Supplementary file 1 [file biomedicines-12-02673-s001.zip › Supplementary Table S2.pdf]

Supplementary Table S2.

**Characteristic**	**Overall**, N = 88	**Pathogenic/likely**, N = 26	**Uncertain significance**, N = 4	**Negative**, N = 58
**Diagnosis**				
aphasia	1	0	0	1
ataxia	34	11	0	23
chorea	5	1	0	4
chorea + dementia	1	0	0	1
dementia	1	0	0	1
dystonia	6	3	0	3
dystonia + spastic syndrome	1	0	0	1
epilepsy	2	1	0	1
leukoencephalopathy	1	0	0	1
leukoencephalopathy + ataxia	4	1	0	3
myoclonus	2	1	0	1
myopathy	1	0	0	1
parkinsonism	21	5	4	12
Progressive Ataxia Palatal Tremor syndrome	1	0	0	1
spastic syndrome	6	3	0	3
tremors	1	0	0	1
**Gene**				
AFG3L2	1	1	0	0
ATP5MC3	3	3	0	0
CACNA1A	3	3	0	0
COL22A1	1	1	0	0
COQ2	1	1	0	0
DNAJC13	1	0	1	0
GCH1	1	1	0	0
GNAO1	1	1	0	0
KCNH1	1	1	0	0
KCNQ2	1	1	0	0
LRRK2	2	2	0	0
OPTN	1	1	0	0
PDE10A	1	0	1	0
POLG	2	2	0	0

**Characteristic**	**Overall**, N = 88	**Pathogenic/likely**, N = 26	**Uncertain significance**, N = 4	**Negative**, N = 58
PRKN	1	1	0	0
RRM2B	1	1	0	0
SACS	1	0	0	1
SCN4A	1	1	0	0
SLC20A	2	0	2	0
SOD1	1	1	0	0
SYNE1	2	2	0	0
TGM6	1	1	0	0
VPS13C	1	1	0	0
Unknown	57	0	0	57
**Zygosity**				
compound heterozygous	3	3	0	—
heterozygous	25	21	4	—
homozygous	1	1	0	—
**Variable type**				
deletion, frame shift	3	3	0	—
duplication, frame shift	1	1	0	—
insertion, frame shift	1	1	0	—
point, missense	22	18	4	—
point, missense, splice donor	1	1	0	—
point, nonsense	2	2	0	—

N, number
